# Feeding and Insurance

**Published:** 1913-04-12

**Authors:** Binnie Dunlop

**Affiliations:** Alexandra Court, S.W.


*To the Editor of* The Hospital.

### Feeding and Insurance.

*To the Editor of* The Hospital.

Sir,—Your interesting note on the report revealing the serious insufficiency of the diet of the labouring classes of Glasgow usefully draws attention to the important fact that "in the last analysis food is the measure of physique." This is borne out by vital statistics which show that falling birth-rates are always accompanied by falling death-rates and will continue to be so until the latter have reached the normal (about 10 per 1,000 per annum, as in New Zealand). Consequently I welcome your suggestion that feeding is much more important than insurance. It seems to me that there are only two honest propositions for the abolition of under-feeding—either (1) that the adult wage-earner should get from the State an additional so many shillings a week for every child he may have, or (2) that the poor should be encouraged to beget no more children than they can themselves afford to maintain properly.—Yours truly,

Binnie Dunlop, M.B., Ch.B.

Alexandra Court, S.W.

### REPLIES TO OUR READERS.

"Interested Reader": We shall be very pleased to consider any contributions on the subject that you may send.—Ed. The Hospital.

